# Dr. Willis Goss Macdonald

**Published:** 1911-02

**Authors:** 


DR. WILLIS GOSS MACDONALD, of Albany, died in the city of his home, December 30, 1910, aged 47 years. He graduated at Albany Medical College in 1887, and soon afterward became instructor in surgery in his Alma Mater. In a few years he was advanced to a professorship in clinical and abdominal surgery, a position that he held at the time of his death. He was also attending surgeon at the Albany hospital and consulting at the Westfield hospital; a member of the State Board of Medical Examiners; a member of the board of managers of the Raybrook State Hospital for the treatment of incipient tuberculosis patients; surgeon of volunteers, with the rank of major during the war with Spain, in charge of the United States Hospital at Fort McPherson, Ga.; also, he was a member of the several local, state and national medical societies, including the American Surgical Association; in 1900 was president of the Medical Society of the State of New York. In his inaugural address before the latter body he spoke thus of the State Board of Medical Examiners. "From information placed at my disposal I am convinced that the work being done by the present board is worthy of the highest commendation, and that the standards established by it are at once the highest and most practical in this country." It is stated that the citizens of Albany contemplate the building of a memorial to perpetuate his name, the nature of which has not yet been determined. Dr. Macdonald's talents were felt in many walks of life, but as a surgeon he was preëminent and the foundation for his knowledge and skill as such was laid while a pupil of Professor A. Vander Veer. Master and pupil early established a friendship which continued in after life most intimately until they were separated by death. Dr. Macdonald was never married. He is survived by aged parents, and one brother. Interment was made at Cobleskill, the place of his nativity.

